# Lymphovascular space invasion and lack of downstaging after neoadjuvant chemotherapy are strong predictors of adverse outcome in young women with locally advanced breast cancer

**DOI:** 10.1002/cam4.586

**Published:** 2015-12-21

**Authors:** Shariq S. Khwaja, Jennifer Ivanovich, Todd A. DeWees, Laura Ochoa, Daniel F. Mullen, Maria Thomas, Julie A. Margenthaler, Amy Cyr, Michael Naughton, Souzan Sanati, Timothy J. Eberlein, William E. Gillanders, Rebecca L. Aft, Jacqueline E. Zoberi, Imran Zoberi

**Affiliations:** ^1^ Department of Radiation OncologyWashington University School of MedicineSt LouisMissouri; ^2^ Department of SurgeryWashington University School of MedicineSt. LouisMissouri; ^3^ Department of MedicineWashington University School of MedicineSt. LouisMissouri; ^4^ Department of Pathology and ImmunologyWashington University School of MedicineSt. LouisMissouri

**Keywords:** Breast cancer, locally advanced, LVSI, neoadjuvant chemotherapy, young women

## Abstract

Younger age diagnosis of breast cancer is a predictor of adverse outcome. Here, we evaluate prognostic factors in young women with locally advanced breast cancer (LABC). We present a retrospective review of 104 patients younger than 40 years with LABC treated with surgery, radiotherapy (RT), and chemotherapy from 2003 to 2014. Patient‐, tumor‐, and treatment‐related factors important for overall survival (OS), local/regional recurrence (LRR), distant metastasis (DM), and recurrence‐free survival (RFS) were evaluated. Mean age at diagnosis was 34 years (23–39 years) with a median follow‐up of 47 months (8–138 months). Breast‐conserving surgery was performed in 27%. Axillary lymph node dissection was performed in 85%. Sixty percent of patients received neoadjuvant chemotherapy with 19% achieving pathologic complete response (pCR), and 61% downstaged. Lymph node positivity was present in 91% and lymphovascular space invasion (LVSI) in 35%. Thirty‐two percent of patients had triple negative tumors (TN, ER‐/PR‐/HER2 nonamplified). Four‐year OS and RFS was 84% and 71%, respectively. Factors associated with worse OS on multivariate analysis include TN status, LVSI, and number of positive lymph nodes. LVSI was also associated with DM and LRR, as well as worse RFS. Downstaging was associated with improved 4 year RFS in patients receiving neoadjuvant chemotherapy (74% vs. 38%, *P* = 0.002). With high risks of recurrence and inferior OS compared to older women, breast cancer in young women can be difficult to treat. Among additional factors, presence of LVSI and lack of downstaging portends a particularly worse prognosis.

## Introduction

In 2015, an estimated 231,840 new cases of invasive breast cancer was diagnosed among women, with approximately 40,000 women expected to have died from their disease 1. Of the total number of new cases, an estimated 1.8% will be in patients under the age of 35. Of the estimated deaths in 2015, approximately 1.0% was estimated in these young women. Cancer is the leading cause of death in women 20–39 years of age, with breast cancer being the most common cancer type in this age group 2. Although the exact definition of “young” women with breast cancer is debatable as evidenced by the variability in defining this population in breast cancer reviews, majority of series suggest a worse prognosis in young women compared with older women 3.

Most published series on breast cancer in young women have included both early‐stage disease with the more aggressive locally advanced breast cancer (LABC). LABC refers to most advanced‐stage nonmetastatic breast tumors 4 and may be operationally defined as tumors greater than 5 cm or that involve the skin or chest wall, or involvement of lymph nodes. Thus, all stage IIIA‐C and stage IIB (T2N1, T3N0) disease would be classified as LABC. Treatment of LABC requires a multimodal approach with a combination of systemic chemotherapy, surgery, and radiation therapy in order to achieve optimal chance of cure. Young women with breast cancer are more likely to have larger tumors and nodal involvement 5, 6. Several factors have been linked to the poor prognosis associated with breast cancer in young women, including large tumor size, higher tumor grade, mitotic rate, lymphovascular space invasion (LVSI), increased expression of HER2, and lower estrogen and progesterone receptor expression 7, 8.

Here, we present our institutional experience in women younger than 40 years of age diagnosed with LABC (T3‐4N0, T1‐4N1) who had radiation as a component of their treatment. Young women with LABC are invariably treated with surgery, radiation therapy, chemotherapy and/or hormonal therapy. These patients are often faced with personal, family, professional, and quality‐of‐life issues that further complicate treatment decision‐making. Many have argued for more tailored therapies in this population arguing that reliance on information from older women will not be adequate to treat younger patients 9. Few studies have exclusively evaluated factors important for survival in young women diagnosed with LABC. We present one of the few series exclusively addressing patient‐, tumor‐, and treatment‐related factors important for overall survival (OS) and recurrence‐free survival (RFS) in this young population with locally advanced disease.

## Materials and Methods

The source for this analysis was de‐identified data from an IRB‐approved prospective registry. From this registry, we conducted a retrospective chart‐based review of 104 breast cancer patients less than 40 years of age treated with a combination of surgery, comprehensive radiotherapy (RT) +/− chemotherapy from 2003 to 2014. Patient‐, tumor‐, and treatment‐specific factors that were hypothesized to predict OS, local/regional recurrence (LRR), distant metastasis (DM), or RFS were evaluated (Table 1). RFS was defined as freedom from local/regional and distant failure.

**Table 1:** Patient and treatment characteristics (*n* = 104)

	*N*	(%)
Mean age at diagnosis (years)	34.5 (23–39)	
Race		
Caucasian	64	61.5
African American	31	29.8
Other	9	8.6
Family history		
Any positive family history	45	43.3
1st degree relative	18/45	40.0
2nd/3rd degree relative	25/45	55.6
Distant relative (>3rd degree)	2/45	4.4
BRCA1/BRCA 2 gene testing		
Testing performed	57	54.8
Mutation identified	10	18.5
No mutation identified	44	77.2
Variant (VUS) identified	3	5.6
No testing performed	47	45.2
Surgery		
BCT	28	26.9
Unilateral Mastectomy	28	26.9
Bilateral Mastectomy	48	46.2
Positive/Close Margin	17	16.4
ALND	88	84.6
Chemotherapy		
Neoadjuvant only	62	59.6
Neoadjuvant+adjuvant	26	25.0
Pathologic CR	12/62	19.4
Downstaged	38/62	61.3
Adjuvant only	34	32.7
Hormonal therapy		
Neoadjuvant	6	5.8
Adjuvant	53	51.0
Radiation therapy		
Median dose (cGy)	5040	
Boost received	53	51.0
Comprehensive RT	99	95.2
Conventional radiation	41	39.4
Helical IMRT	12	11.5
Nonhelical IMRT	51	49.0

BCT, breast conserving therapy; IMRT, intensity modulated radiation therapy; ALND, axillary lymph node dissection; CR, complete response; VUS, variant of unknown significance.

Only patients with LABC were included. LABC was operationally defined as any node positive or T3‐4N0 cancer. For patients receiving neoadjuvant therapy, pretreatment clinical stage was utilized. All patients clinically node negative had an axillary ultrasound (US) and subsequent fine needle aspiration (FNA) or sentinel lymph node biopsy performed for suspicious nodes. All clinically node positive patients had FNA confirmation. T3N0 patients who received mastectomy followed by postmastectomy radiation treatment had one or more of high risk features warranting radiation treatment, which included LVSI, close (<2 mm) or positive margin, and/or triple negative (TN) receptor status.

Patient‐specific factors recorded include: age at diagnosis, race, BRCA1 and BRCA2 gene mutation status, and family history of breast cancer. Tumor‐specific factors recorded include biomarker status (estrogen receptor (ER), progesterone receptor (PR), and HER2), multifocality, tumor size, number of lymph nodes involved with disease (LN), extranodal extension (ENE), perineural invasion (PNI), and LVSI. LVSI was documented as either present or absent. LVSI was not consistently noted as extensive or focal in pathology reports. Treatment‐specific factors recorded include type of surgery (breast conserving surgery versus mastectomy, type of axillary surgery), type of neoadjuvant and/or adjuvant systemic therapy, achievement of pathologic complete response (pCR), downstaging, radiation technique, dose, and whether a boost to the scar was given. Downstaging was defined as any decrease in T stage or N stage after neoadjuvant therapy. Patients with a decrease in N stage but with an increase in T stage (and vice versa) were not classified as downstaged. Here pCR was defined as no evidence of in situ or invasive disease after neoadjuvant therapy. All patients received comprehensive radiation therapy, which included coverage of the breast or chest wall, axillary, supraclavicular and internal mammary lymph nodes, except patients with T3N0 cancers, who received chest wall radiation alone.

Univariate and multivariate COX regression was performed for OS, LR, DM, and RFS. Survival was assessed by Kaplan–Meier analyses utilizing the log‐rank test for statistical significance. A *P*‐value <0.05 was considered statistically significant.

## Results

### Patient and treatment characteristics

Patient and treatment characteristics are outlined in Table 1. Median follow‐up time was 47 months (range 8–138 months). The mean age at diagnosis was 34.5 years (range 23–39 years). The majority of patients were Caucasian (61.5%) or African American (29.8%). Fifty‐seven (54.8%) women underwent BRCA1 and BRCA2 gene testing. Of those tested, 10 women (18.5%) had a mutation and three women had a variant of unknown clinical significance (VUS). The remaining women tested negative. Forty‐five patients (43.3%) were documented to have any positive family history of breast cancer, with 18 patients (40.0%) having a documented first degree relative and 25 patients (55.6% with a second/third degree relative. Twenty‐eight patients (26.9%) had breast‐conserving surgery, 28 patients (26.9%) had a unilateral mastectomy, and 48 patients (46.2%) had bilateral mastectomies. Surgery included an axillary lymph node dissection in 88 patients (84.6%) with a median number of lymph nodes (LN) removed of 15 nodes (interquartile range 10–18). The remaining patients had a sentinel lymph node excision. A close (<2 mm) or positive margin was observed in 17 patients (16.4%), 6 occurring after breast‐conservation, and the remaining 11 after mastectomy.

A total of 62 patients (59.6%) were administered neoadjuvant treatment (chemotherapy with or without targeted therapy) with 12 of these patients (19.4%) showing a pathologic complete response to treatment. A total of 38 of the 62 patients (61.3%) had downstaging of their tumors. Thirty‐four patients (32.7%) received adjuvant chemotherapy alone, whereas 26 patients (25.0%) received both neoadjuvant and adjuvant chemotherapy. Various chemotherapy regimens were utilized. The most common was AC plus or minus T (doxorubicin, cyclophosphamide, taxol), FEC (5‐fluoruracil, epirubicin, cyclophosphamide), and TAC (docetaxel, doxorubicin, cyclophosphamide). Select patients also received Avastin (bevacizumab) or Herceptin (trastuzumab). A total of six patients (5.9%) received neoadjuvant hormonal therapy with an aromatase inhibitor or tamoxifen. Fifty‐three patients (51.0%) received adjuvant hormonal therapy with the most common treatment being tamoxifen (*n* = 40, 74.1%). All patients received adjuvant radiation therapy, which was delivered using conventional fields in 41 patients (39.4%), helical intensity modulated radiation therapy (H‐IMRT) in 12 patients (11.4%), and nonhelical IMRT in 51 patients (49.0%), for which simulation and treatment techniques have been described previously 10. The median dose was 5040 cGy. The vast majority of patients received comprehensive radiotherapy (*n* = 99, 95.2%) comprising chest wall and regional nodal volume radiation, including the internal mammary nodes. The remaining five patients received chest wall radiation alone. These patients had T3N0 disease. Fifty‐three patients (51.0%) received a boost, with a median boost dose of 1000 cGy (range 600–2000 cGy).

### Tumor characteristics

Tumor‐specific characteristics are outlined in Table 2. The mean tumor size was 3.2 cm. Estrogen‐receptor positive (ER+)/Progesterone‐receptor positive (PR+)/HER2 nonamplified tumors comprised 34%, ER−/PR−/HER2 amplified tumors 14%, ER+/PR+/HER2 amplified 11% and triple negative (TN, ER−/PR−/HER2 nonamplified) 32%. The overwhelming majority (96.2%) of tumors were invasive ductal carcinomas (IDC). Majority of tumors were grade III (*n* = 54, 60.7%) or grade II (*n* = 27, 30.3%). Tumors were largely stage II (*n* = 40, 38.5%) or stage III (*n* = 63, 60.6%). A clinical or pathologic T4 was observed in 14 patients (13.5%). The overwhelming majority of patients were LN positive (*n* = 95, 91.3%). Pathologic LN positivity, as assessed by sentinel LN biopsy or axillary LN dissection, was present in 76 patients (73.1%). Fourteen patients (13.3%) were pathologically LN negative, but were observed to have pathologic LN positivity on pretreatment ultrasound FNA. LVSI was seen in 36 patients (34.6%), ENE was seen in 27 patients (26.0%), and PNI in 6 patients (5.8%). Multifocal disease was seen in 20 patients (19.2%).

**Table 2:** Tumor characteristics

	*N*	% (of total)
Mean size (cm) (range in cm)	3.2 (0.2–23.0)	
Luminal A (ER+/PR+/HER2 nonamp G1‐2)	20	19.2
Luminal B (ER+/PR+/HER2 nonamp G3)	15	14.4
HER2 (ER−/PR−/HER2 amp)	14	13.5
Luminal‐HER2 (ER+/PR+/HER2 amp)	11	10.6
Triple Negative (ER−/PR−/HER2 nonamp)	33	31.7
Histology		
Invasive ductal	101	96.2
Invasive lobular	2	1.9
Pathologic grade		
I	8	7.7
II	27	26.0
III	53	51.0
Stage (clinical or pathologic)		
II	40	38.5
III	63	60.6
Clinical or pathologic T4	14	13.5
Lymph node positive	95	91.3
Path. positive LN (SLNB or AXLND)	76	73.1
Path. LN negative but LN+ at US	14	13.3
Path. LN negative and LN− at US	5	4.8
LVSI	36	34.6
ENE	27	26.0
PNI	6	5.8
Multifocal	20	19.2

LN, lymph nodes; LVSI, lymphovascular space invasion; ENE, extranodal extension; PNI, perineural invasion, ER, estrogen receptor; PR, progresterone receptor; amp, amplified.

### Events

Table 3 outlines the number of deaths, LRRs, and sites of metastatic disease. With a median follow‐up of 47 months (range 8–138 months), 16 patients (15.4%) died of their disease. Twelve patients had local recurrences either in‐breast, nodal, or chest wall (11.4%). Only 1 of 12 patients had an in‐breast recurrence only. Four patients had nodal and chest wall recurrences. Of the remaining LRRs, 6 patients had a nodal only recurrence and one patient had both a nodal and in‐breast recurrence. All 12 of these patients eventually developed distant metastatic disease. In total, 29 patients (27.9%) had distant recurrences. Some patients had more than one site involved. Most common sites were brain, bone, lung, and liver. Recurrences were treated with chemotherapy only (*n* = 10), radiotherapy only (*n* = 7), or combination chemotherapy and radiotherapy (*n* = 10). The remaining two patients chose pain control with oral analgesics. To date there have been no treatment‐induced malignancies.

**Table 3:** Summary of events (*n* = 104 patients)

	*N*	(%)
Median follow‐up in months (range)	47 (8–138)	
Number of deaths	16	15.4
Patients with recurrence	29	27.9
Local/regional recurrences		
Total	12	11.4
In‐breast only	1/12	8.3
Nodal only	6/12	50.0
Nodal and in‐breast	1/12	8.3
Nodal and chest wall	4/12	33.3
Patients with distant recurrences		
Total	29	27.9
Brain	11/29	37.9
Bone	16/29	55.2
Lung	12/29	41.3
Liver	14/29	48.3
Other	10/29	34.5

Univariate and multivariate (MVA) Cox regressions were performed on the patient‐, tumor‐, and treatment‐specific factors (Table 4). Univariate analysis revealed that ER positivity was associated with better OS, whereas LVSI, number of positive LN, and TN were associated with poor OS (*P* < 0.05). LVSI, number of positive LN, TN status, and ER positivity continued to show significance on MVA. Similar analyses were performed for local/regional failure‐free survival (LRRF), DM‐free survival, and RFS. Univariate analysis for LRRF revealed that African American race, number of positive LN, and LVSI were associated with poor LRRF, whereas downstaging was associated with better LRRF (*P* < 0.05). Number of positive LN, African American race, and LVSI remained significant on MVA. Factors associated with better DM‐free survival on univariate analysis included downstaging and positive family history of breast cancer, whereas LVSI, number of positive LN, and African American race were associated with poor DM‐free survival (*P* < 0.05). On MVA, LVSI, and downstaging remained significant in the model. For RFS, univariate results were similar to those in the DM‐free survival analysis, with LVSI and downstaging remaining significant on MVA (*P* < 0.05). In the patient cohort receiving neoadjuvant chemotherapy, LVSI and downstaging were significant factors for both RFS and DM‐free survival. For these patients, number of positive LN was also a significant factor for LRRF on MVA (*P* < 0.014).

**Table 4:** Univariate/multivariate COX regression

Parameter	Univariate			Multivariate		
	HR	95% CI	*P*‐value	HR	95% CI	*P*‐value
Overall survival						
Triple negative	2.867	1.073–7.662	0.036	6.421	2.009–20.525	0.002
LVSI	3.619	1.312–9.983	0.013	3.324	1.190–9.282	0.022
Number of positive LN's	1.134	1.041–1.235	0.004	1.192	1.077–1.318	0.001
ER‐positive	0.344	0.119–0.992	0.048	0.159	0.050–0.508	0.002
Any recurrence						
LVSI	2.616	1.252–5.464	0.011	2.452	1.165–5.160	0.018
Downstaged	0.268	0.108–0.665	0.005	0.340	0.128–0.907	0.031
African‐American race	2.383	1.143–4.970	0.021			
Positive family history	0.428	0.183–1.004	0.051			
Number of positive LN's	1.098	1.017–1.187	0.018			
Locoregional recurrence						
African‐American Race	3.479	1.103–10.973	0.033	4.270	1.279–14.250	0.018
LVSI	3.821	1.113–13.122	0.033	4.283	1.141–16.072	0.031
Number of Positive LN's	1.194	1.090–1.307	<0.001	1.203	1.093–1.325	<0.001
Downstaged	0.179	0.034–0.938	0.042			
Distant metastasis						
LVSI	2.525	1.210–5.268	0.014	2.441	1.161–5.131	0.019
Downstaged	0.285	0.116–0.704	0.006	0.340	0.128–0.907	0.031
African‐American race	2.374	1.138–4.951	0.021			
Positive family history	0.424	0.181–0.994	0.048			
Number of positive LN's	1.095	1.015–1.181	0.019			

TN, triple negative; ALND, axillary lymph node dissection; LVSI, lymphovascular space invasion; LN, lymph nodes; ER, estrogen receptor; HR, hazard ratio (HR > 1 represents increased likelihood of having event).

**Figure 1:**
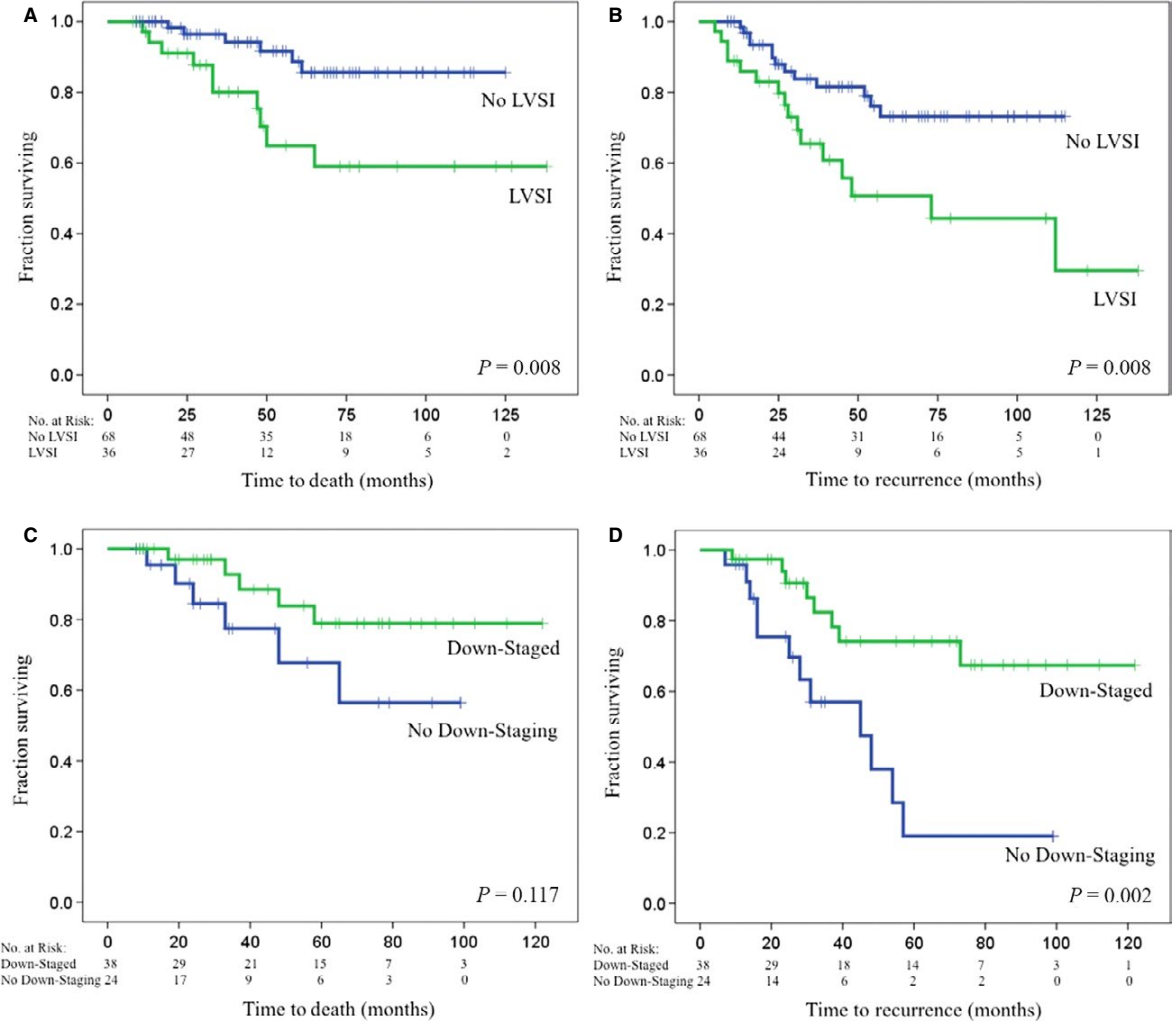
Overall survival and recurrence‐free survival (RFS). Kaplan–Meier survival plots of (A) overall survival (OS) with and without lymphovascular space invasion (LVSI), (B) RFS with and without LVSI, (C) OS in patients receiving neoadjuvant chemotherapy stratified by downstaging, (D) RFS in patients receiving neoadjuvant chemotherapy stratified by downstaging. *P*‐values represent results of log‐rank analysis.

Table 5 presents survival statistics for OS, RFS, LRRF, and DM – free survival. OS at 2 and 4 years was 95% and 84%, respectively. RFS at 2 and 4 years was 86% and 71%, respectively. LRRF at 2 and 4 years was 95% and 87%, respectively. DM‐free survival at 2 and 4 years was 87% and 72%, respectively. Kaplan–Meier survival curves were generated using LVSI status for OS and RFS (Fig. 1A and B, respectively). Four‐year OS for patients that had no evidence of LVSI was 92%, whereas patients with documented LVSI had a 70% 4‐year OS (*P* = 0.009 on log‐rank). Similarly, 4‐year RFS for patients without LVSI was 82% as opposed to 52% in patients with LVSI (*P* = 0.010 on log‐rank). The impact of downstaging on OS and RFS in patients receiving neoadjuvant chemotherapy is graphically represented in Figure 1C and D, respectively. Patients successfully downstaged after neoadjuvant chemotherapy had improved RFS (*P* = 0.002), but this did not affect their OS (*P* = 0.117).

**Table 5:** Impact of LVSI on outcomes

Survival	2 years (%)	4 years (%)	*P*‐value
Overall survival	95	84	0.009
With LVSI	91	70	
Without LVSI	96	92	
Recurrence‐free survival (RFS)	86	71	0.010
With LVSI	80	52	
Without LVSI	88	82	
Locoregional RFS	95	87	0.022
With LVSI	91	73	
Without LVSI	97	95	
Distant metastasis‐free survival	87	72	0.010
With LVSI	86	54	
Without LVSI	88	82	

*P*‐values calculated using Log‐rank (Mantel‐Cox). LVSI, lymphovascular space invasion.

## Discussion

The present series represents one of the few studies exploring factors associated with survival exclusively in young women with LABC. Younger age has been shown in multiple retrospective studies and subset analyses of larger randomized trials to be a powerful adverse prognostic indicator of LRR and distant metastases in breast cancer patients. Many of these randomized trials, however, did not have adequate representation of women in this young age group. Investigators in the UK recently published a prospective study (The Prospective Study of Outcomes in Sporadic and Hereditary Breast Cancer – POSH) designed to investigate factors affecting prognosis in this group 11. As one of the few prospective studies, POSH highlights the relative poor prognosis in women less than 40 years, with an OS at 5 years of 82% and DM rate of 24%. Previous retrospective analyses and the aforementioned prospective trial in young women have included early stage breast cancer patients 8, 12, 13, 14, 15, 16, 17, 18. With the understanding that young women tend to present with more advanced disease, our goal in limiting this study to age less than 40 with LABC was to remove age as a variable and focus solely on those patients who presented with locally advanced disease.

In presenting a pooled analysis of four European Organization for Research and Treatment of Cancer (EORTC) clinical trials, van der Hage et al. reveal tumor size, nodal status, and molecular subtype as independent prognostic factors for OS in young women with breast cancer, with only molecular subtype as a prognostic factor for OS in the node negative group 19. In our study of predominantly node positive patients, TN status and presence of LVSI were shown on multivariate analysis to be predictive of OS. Number of positive lymph nodes also correlated with worse OS. Patients presenting with LVSI had an absolute decrease in survival at 4 years of over 20%. Type of surgery (breast conserving therapy [BCT] vs mastectomy) in this young age group did not affect any of the outcomes measured. This is consistent with a recently published meta‐analysis summarizing 6 studies comparing OS between patients receiving BCT vs mastectomy in patients less than 40 years 20.

In terms of LRR‐free survival, distant‐metastasis free‐survival and RFS, presence of LVSI was again highlighted as a variable significant on multivariate analysis. Our data demonstrate a DM‐free survival of only 54% at 4 years in young women presenting with LVSI. In a recent study of early stage breast cancer, Freedman et al. studied the prognostic importance of LVSI after conservative surgery and radiation 21. Although 5‐ and 10‐year locoregional RFS and OS were decreased for patients with LVSI in their cohort of patients with a median age around 55, LVSI was not found to be an independent predictor of local/regional failure or decreased survival on multivariate analysis. In our cohort of LABC, young women with LVSI were more likely to recur, having a RFS at four years of approximately 52% compared to 82% for young women without evidence of LVSI. Huang et al. investigated predictors of LRR in patients with a median age of 50 with LABC treated with neoadjuvant chemotherapy, mastectomy, and radiotherapy, revealing factors on multivariate analysis that independently predicted for LRR: skin/nipple involvement, supraclavicular disease, extracapsular extension, and estrogen‐receptor negative disease 22. Our series reveals a number of positive LN's, African American race, and presence of LVSI as significant factors for LRR.

There have been significant efforts to elucidate an underlying biological explanation as to why young women have a more aggressive course compared to elderly women. Recent gene expression profiling studies have showed that young women have a higher probability of PI3K, Myc, and B‐catenin deregulation and lower mRNA levels of ER and progesterone receptor, but higher levels of HER2 and epidermal growth factor receptor 23. Azim et al. evaluated whether previously published proliferation, stroma, and immune‐related gene signatures were predictive of prognosis in young women 24. Their analyses suggested that breast cancer in young women is enriched with processes related to mammary stem cells (e.g., RANKL, c‐kit, luminal progenitors) and growth factor signaling. These studies and others like it have successfully identified certain signaling pathways and gene expression signatures associated with breast cancer in young women, suggesting that breast cancer in young women is a unique disease entity compared to breast cancer arising in the older population.

It is becoming clear that achieving a pathologic complete response (pCR) prior to surgery improves disease‐free survival 25. Loibl and colleagues of the German Breast Group presented results of a German meta‐analysis at the 2012 San Antonio Breast Cancer Symposium. Interestingly, they found that the pathologic complete response was higher in young women compared to older women (23% vs. 13.5%). Furthermore, young women who did not achieve a pCR had inferior disease‐free and local RFS compared to those women that achieved pCR. In our series, patients with TN disease were more likely to achieve a pCR compared to non‐TN patients (*P* = 0.04), consistent with a study from MD Anderson 26. Cortazar and colleagues in a recent Lancet article showed that patients with pCR had improved survival, but the utility of pCR as a surrogate endpoint for survival was not validated 27. A recent meta‐analysis of 29 neoadjuvant trials reached the conclusion that use of pCR as a surrogate for outcomes is not justified 28. Regardless of the utility in using pCR to predict outcomes, the unique biology of breast cancer in young women may render their tumors more responsive to neoadjuvant treatment. In our cohort of young women with LABC, 12 patients (19.4%) achieved a pCR and 38 patients (61.3%) were downstaged, which was a statistically significant factor for DM‐free survival and RFS. However, the high rate of pCR/downstaging may be related to the high proportion of triple negative patients in this series. Moreover, downstaging after neoadjuvant chemotherapy was associated with RFS but not OS, likely due to short follow‐up and salvage therapy in ER+ patients.

There are inherent limitations associated with a single institution retrospective analysis. It is understood that treatment regimens at institutions can vary significantly. It should be emphasized that this series only included patients that had radiotherapy as a required component of management. However, LABC in young women is invariably treated with some combination of surgery, radiation, and chemotherapy.

This study highlights two already known and established poor prognostic indicators, namely LVSI and poor response to neoadjuvant chemotherapy, in the overall management of these young women. Given an inferior prognosis compared to older women and evidence of a biologically distinct entity, there appears to be a need for clinical trials geared specifically to young women with breast cancer. Prospectively designed collaborations to specifically investigate novel therapeutic approaches in young women with breast cancer are required.

## Conflict of Interest

The authors declare no conflicts of interest.
